# Novel Action Targets of Natural Product Gliotoxin in Photosynthetic Apparatus

**DOI:** 10.3389/fpls.2019.01688

**Published:** 2020-01-17

**Authors:** Yanjing Guo, Jing Cheng, Yuping Lu, He Wang, Yazhi Gao, Jiale Shi, Cancan Yin, Xiaoxiong Wang, Shiguo Chen, Reto Jörg Strasser, Sheng Qiang

**Affiliations:** ^1^ Weed Research Laboratory, Nanjing Agricultural University, Nanjing, China; ^2^ Bioenergetics Laboratory, University of Geneva, Geneva, Switzerland

**Keywords:** chlorophyll *a* fluorescence (OJIP) transient, mycotoxin, action target, D1 protein, binding model

## Abstract

Gliotoxin (GT) is a fungal secondary metabolite that has attracted great interest due to its high biological activity since it was discovered by the 1930s. It exhibits a unique structure that contains a N-C = O group as the characteristics of the classical PSII inhibitor. However, GT’s phytotoxicity, herbicidal activity and primary action targets in plants remain hidden. Here, it is found that GT can cause brown or white leaf spot of various monocotyledonous and dicotyledonous plants, being regarded as a potential herbicidal agent. The multiple sites of GT action are located in two photosystems. GT decreases the rate of oxygen evolution of PSII with an *I*_50_ value of 60 µM. Chlorophyll fluorescence data from *Chlamydomonas reinhardtii* cells and spinach thylakoids implicate that GT affects both PSII electron transport at the acceptor side and the reduction rate of PSI end electron acceptors’ pool. The major direct action target of GT is the plastoquinone Q_B_-site of the D1 protein in PSII, where GT inserts in the Q_B_ binding niche by replacing native plastoquinone (PQ) and then interrupts electron flow beyond plastoquinone Q_A_. This leads to severe inactivation of PSII RCs and a significant decrease of PSII overall photosynthetic activity. Based on the simulated modeling of GT docking to the D1 protein of spinach, it is proposed that GT binds to the-Q_B_-site through two hydrogen bonds between GT and D1-Ser264 and D1-His252. A hydrogen bond is formed between the aromatic hydroxyl oxygen of GT and the residue Ser264 in the D1 protein. The 4-carbonyl group of GT provides another hydrogen bond to the residue D1-His252. So, it is concluded that GT is a novel natural PSII inhibitor. In the future, GT may have the potential for development into a bioherbicide or being utilized as a lead compound to design more new derivatives.

## Introduction

Gliotoxin (GT), an alkaloid with a molecular mass of 326 Da, is the most important and well-known epipolythiodioxypipeazine (ETP)-type mycotoxin with biological active internal disulfide bridge (Smith et al., 2016). Since it was discovered by the 1930s, GT has been isolated from various fungal species, including *Trichoderma*, *Aspergillus fumigatus*, *Eurotium chevalieri*, *Neosartorya pseudofischeri*, some *Penicillium* spp., and *Acremonium* spp. Numerous studies show that GT processes medicinal properties, including immunosuppressive, antitumour, antibacterial, and antiviral activity. However, it was discarded from clinical practice for its toxicity. GT is also recognized for an antibiotic substance involved in biological control of plant disease because it can cause cytoplasmic leakage, inhibit the germination of sporangia and mycelia growth of some plant pathogenic fungi (Scharf et al., 2016). Several GT-producing strains of *Trichoderma virens* have been successfully commercialized as biopesticides and widely used in agriculture (Lumsden and Walter, 2003; Khan et al., 2011).

Previous references indicated that GT has multiple cellular effects because of its different action targets. Early in 1968, it was found that GT can prevent viral RNA replication due to the specific inhibition of reverse transcriptase (Miller et al., 1968). In eukaryotic cells, GT has been proven as inhibitor of several enzymes such as farnesyltransferase, geranylgeranyltransferase, nicotinamide adenine dinucleotide phosphate (NADPH) oxidase, alcohol-dehydrogenases, and nuclear factor-kappaB, causing apoptosis and necrosis in various cell types (Vigushin et al., 2004; Kim and Park, 2016; Scharf et al., 2016; Arias et al., 2018). Further evidence revealed that necrotic cell death induced by GT in murine thymocytes is associated with activation of a redox active calcium channel in the plasma membrane (Hurne et al., 2002). The inhibition of proteasome activity is one of the putative molecular targets of GT-mediated apoptosis in immune cells (Kroll et al., 1999; Dolan et al., 2015; Li et al., 2018). Based on the fact that the disulfide bridge of GT allows the cross linking with proteins and generates reactive oxygen species (ROS) through the redox cycling between reduced and oxidized forms, ROS is believed to be also responsible for DNA damage and apoptosis in cells of immune system (Harms et al., 2015; Nouri et al., 2015). Additionally, it is proposed that GT can perturb microfilament structure and induce cell detachment (Jordan and Pedersen, 1986). Recent work demonstrated that GT can target integrins to induce anoikis on lung epithelial cells (Haun et al., 2018).

However, at present very little attention is paid to the phytotoxicity of GT. It was reported that GT is inhibitory to root growth of clover and mustard accompanied by reduction in percentage germination of seeds (Wright, 1951). Similarly, GT shows potent growth inhibition against lettuce seedlings (Furuta et al., 1984; Haraguchi et al., 1992). Haraguchi et al. (1996; 1997) discovered that GT inhibits growth of cultured tobacco cells and pea seedling roots through the interference with the biosynthesis of branched-chain amino acids by reducing acetolactate synthase (ALS) activity. ALS is one of the targets of commercial herbicides. This means GT is possibly used to develop directly as a potential bioherbicide or design more novel derivatives as a template in the future. However, GT’s herbicidal activity, multiple primary action targets, and mechanistic details of certain physiological effects on plants are unclear.

The goal of this study is to evaluate the herbicidal activity of GT, probe its action targets on two photosystems, and tests two hypotheses as following. Firstly, GT can cause leaf lesion of various plant species, possessing excellent herbicidal activity. Secondly, GT is a novel natural photosynthetic inhibitor, decreasing PSII activity by binding to D1 protein. To prove these hypotheses, here phytotoxicity of GT to 10 different plant species was determined, and then chlorophyll (Chl) *a* fluorescence technique as an expeditious tool was utilized to identify and localize effects of GT on two photosystems. Finally, based on the structural information available from atrazine- and DCMU- binding to the reaction center of purple bacteria, a simulated modeling of GT interacting with the reaction center of spinach was constructed. Identification of the detailed molecular action targets of GT may help to design high-affinity GT-based derivatives, which is important for developing future new bio-based herbicides.

## Materials and Methods

### Plants and Chemicals

Ten species of plants (**Table 1**) were cultured in soil from seed for about 2 months in the greenhouse at 20–25 °C and illuminated for 12 h with approximate 200 µmol m^−2^s^−1^ white light.

**Table 1:** Formulae and explanation of the technical data of the OJIP curves and the selected JIP-test parameters used in this study^a^.

**Technical fluorescence parameters**	
F_t_	fluorescence at time t after onset of actinic illumination
F_O_ ≅ F_20µs_	minimal fluorescence, when all PSII RCs are open
F_L_ ≡ F_150µs_	fluorescence intensity at the L-step (150 µs) of OJIP
F_K_ ≡ F_300µs_	fluorescence intensity at the K-step (300 µs) of OJIP
F_J_ ≡ F_2ms_	fluorescence intensity at the J-step (2 ms) of OJIP
F_I_ ≡ F_30ms_	fluorescence intensity at the I-step (30 ms) of OJIP
F_P_ (= F_M_)	maximal recorded fluorescence intensity, at the peak P of OJIP
F_v_ ≡ F_t_ – F_O_	variable fluorescence at time t
F_V_ ≡ F_M_ – F_O_	maximal variable fluorescence
t_FM_	time (in ms) to reach the maximal fluorescence intensity F_M_
V_t_ ≡ (F_t_ – F_O_)/(F_M_ – F_O_)	relative variable fluorescence at time t
V_K_ = (F_K_ – F_O_)/(F_M_ – F_O_)	relative variable fluorescence at the K-step
V_J_ = (F_J_ – F_O_)/(F_M_ – F_O_)	relative variable fluorescence at the J-step
W_t_ ≡ (F_t_ – F_O_)/(F_J_ – F_O_)	relative variable fluorescence F_v_ to the amplitude F_J_ – F_O_
W_OK_ = (F_t_ – F_O_)/(F_K_ – F_O_)	ratio of variable fluorescence F_t_ – F_O_ to the amplitude F_K_ – F_O_
W_OJ_ = (F_t_ – F_O_)/(F_J_ – F_O_)	ratio of variable fluorescence F_t_ – F_O_ to the amplitude F_J_ – F_O_
W_OI_ = (F_t_ – F_O_)/(F_I_ – F_O_)	ratio of variable fluorescence F_t_ – F_O_ to the amplitude F_I_ – F_O_
W_IP_ = (F_t_ – F_I_)/(F_P_ – F_I_)	ratio of variable fluorescence F_t_ – F_I_ to the amplitude F_P_– F_I_
M_0_ ≡ 4(F_270μs_ – F_O_)/(F_M_ – F_O_)	approximated initial slope (in ms^–1^) of the fluorescence transient normalized on the maximal variable fluorescence F_V_
S_m_ ≡ Area/(F_M_ – F_O_)	normalized total complementary area above the O-J-I-P transient (reflecting multiple-turnover Q_A_ reduction events)
Ss = V_J_/M_0_	normalized total complementary area corresponding only to the O-J phase (reflecting single-turnover Q_A_ reduction events)
**Quantum efficiencies or flux ratios**	
φ_Po_ = PHI(P_0_) = TR_0_/ABS = 1– F_O_/F_M_	maximum quantum yield for primary photochemistry
ψ_Eo_ = PSI_0_ = ET_0_/TR_0_ = 1–V_J_	probability that an electron moves further than ${\text{Q}}_{\text{A}}^{-}$
φ_Eo_ = PHI(E_0_) = ET_0_/ABS = (1– F_O_/F_M_) (1–V_J_)	quantum yield for electron transport (ET)
φ_Do_ = PHI(D_0_) = 1- φ_Po_ = F_O_/F_M_	quantum yield (at t = 0) of energy dissipation
φ_Ro_ = RE_0_/ABS = φ_Po._ ψ_Eo_. δ_Ro_ = φ_Po_. (1–V_I_)	quantum yield for reduction of the end electron acceptors at the PSI acceptor side (RE)
δ_Ro_ = RE_0_/ET_0_ = (1 – V_I_)/(1 – V_J_)	probability that an electron is transported from the reduced intersystem electron acceptors to the final electron acceptors of PSI
γ_RC_ = Chl_RC_/Chl_total_ = RC/(ABS+RC)	probability that a PSII Chl molecule functions as RC
**Phenomenological energy fluxes (per excited leaf cross-section-CS)**	
ABS/CS = Chl/CS	absorption flux per CS
TR_0_/CS = φ_Po_. (ABS/CS)	trapped energy flux per CS
ET_0_/CS = φ_Po_. ψ_Eo_. (ABS/CS)	electron transport flux per CS
**Density of RCs**	
RC/CS = φ_Po_. (V_J_/M_0_). (ABS/CS)	Q_A_-reducing RCs per CS
Q_A_-reducing centers = (RC/RC_reference_).(ABS/ABS_reference_) = [(RC/CS)_treatment_/(RC/CS)_control_]. [(ABS/CS)_treatment_/(ABS/CS)_control_]	The fraction of Q_A_-reducing reaction centers
Non-Q_A_-reducing centers = 1- Q_A_-reducing centers	The fraction of non-Q_A_-reducing reaction centers, also so-called heat sink centers or silent centers
S_m_/t_FM_ = [RC_open_/(RC_close_ + RC_open_)]av = [Q_A_/Q_A(total)_]av	average fraction of open RCs of PSII in the time span between 0 to *t*_*F*_*M*__
R_J_ = [ψ_Eo (control)_ − ψ_Eo (treatment)_]/ψ_Eo (control)_ = [V_J (treatment)_ – V_J (control)_]/[1 − V_J (control)_]	number of PSII RCs with Q_B_-site filled by PSII inhibitor
**Performance indexes**	
$PI_{ABS}\equiv \frac{\gamma_{RC}}{1-\gamma_{RC}}\cdot \frac{\varphi_{Po}}{1-\varphi_{Po}}\cdot \frac{\psi_{Eo}}{1-\psi_{Eo}}$	performance index (potential) for energy conservation from photons absorbed by PSII to the reduction of intersystem electron acceptors
$PI_{total}\equiv PI_{ABS}\cdot \frac{\delta_{Ro}}{1-\delta_{Ro}}$	performance index (potential) for energy conservation from photons absorbed by PSII to the reduction of PSI end acceptors

a Subscript “0” (or “o” when written after another subscript) indicates that the parameter refers to the onset of illumination, when all RCs are assumed to be open.

The green alga, *Chlamydomonas reinhardtii*, was obtained from the Freshwater Algae Culture Collection at the Institute of Hydrobiology (FACHB-collection 2221, Chinese Academy of Science, China). Cells were grown at 25°C in liquid Tris-acetate-phosphate medium, shaken 3 to 4 times per day, under about 100 µmol m^−2^ s^−1^ white light (day/night, 12 h/12 h). The experiments were done with 3-day old cultures during their logarithmic growth phase (Gao et al., 2018).

Gliotoxin (CAS No. 7562-61-0), diuron (CAS No. 330-54-1, DCMU, 3-(3,4-Dichlorophenyl)-1,1-dimethylurea), methyl viologen (CAS No. 75365-73-0, MV, 1,1’-dimethyl-4,4’-bipyridinium-dichloride), and dimethyl sulphoxide (CAS No. 67-68-5, DMSO) were obtained from Sigma-Aldrich, and other common chemical reagents used in this work were purchased from Amresco. The Gliotoxin, DCMU and MV stock solutions were prepared in 100% DMSO and diluted in distilled water as required. The final concentration of DMSO in every experiment was less than 1% (v/v).

### Phytotoxicity Assay

The detached-intact leaves from 10 species of plants were rinsed with sterilized water, subsequently blotted-dry with sterile paper, and then placed in Petri dishes with wet filter paper. Leaves were punctured using a needle from their margin on the abaxial side. A 10 µl of 1% DMSO (mock) or GT solution at different concentrations (100, 500, and 1,000 µM) was dripped onto the punctured wound of leaves. All Petri dishes were placed in a growth chamber for 96 h at 25°C under around 200 μmol m^−2^s^−1^ white light (day/night, 12h/12h). The diameter of leaf lesions was measured with calipers. Each mean value was obtained from at least fifteen leaf samples.

### Measurement of PSII Oxygen Evolution Rate

The rate of oxygen evolution of PSII was measured using a Clark type oxygen electrode (Hansatech Instruments Ltd., King’s Lynn, UK) according to Chen et al. (2007). *C. reinhardtii* cells were resuspended in Buffer A with a 0.65 A_750_, and then GT and DCMU were individually added into 2 ml suspensions with the indicated concentrations. After the cells were incubated for 3 h in darkness at 25°C, treated-cells containing 45 µg chlorophylls were added into the reaction medium including 50 mM Hepes-KOH buffer (7.6), 4 mM K_3_Fe(CN)_6_, 5 mM NH_4_Cl, 1 mM p-Phenylenediamine. Cells were illuminated with 400 µmol photons m^−2^ s^−1^ red actinic light. The rate of oxygen evolution was measured during the first three minutes after onset of illumination.

### Chl *a* Fluorescence Imaging

Chl *a* fluorescence imaging was determined using a pulse-modulated Imaging-PAM M-series fluorometer (MAXI-version, Heinz Walz GmbH, Effeltrich, Germany) in three independent experiments (Gao et al., 2018). *C. reinhardtii* cells were harvested and resuspended in Buffer A (20 mM HEPES-KOH pH 7.5, 350 mM sucrose, and 2.0 mM MgCl_2_) with a 0.65 A_750_. 200 μl of cell suspensions with 1% DMSO (mock), GT (10, 50, and 100 µM) were added into the 96-well black microtiter plate, incubating for 2.5 h under 100 µmol m^−2^ s^−1^ white light at 25°C. Subsequently, the samples were placed under the imaging system camera for 0.5 h dark-adaptation after focusing of the camera. Images of fluorescence were recorded at 0.25 µmol m^−2^ s^−1^ measuring light, 110 µmol m^−2^ s^−1^ actinic light, and 6,000 µmol m^−2^ s^−1^ saturation pulse light. In the absence of actinic illumination, on application of a weak measuring light and saturation pulse, the minimum fluorescence yield (F_O_) and the maximum fluorescence yield (F_M_) were determined respectively, from which the F_V_/F_M_ was calculated. The fluorescence yield (F_S_) was determined after the addition of actinic light. During 315 s of actinic illumination, the maximum fluorescence yield in the light-adapted state (F_M_′) was determined during the repeated saturation pulse light for 0.8 s at intervals of 20 s. The electron transport rate (ETR), effective quantum yield (Yield), and photochemical quenching coefficient (qP) were also calculated automatically based on F_O_, F_M_, F_S_, and F_M_′.

### Chl *a* Fluorescence Rise Kinetics OJIP and the Modulated 820 Nm Reflection (MR_820_)

Chl *a* fluorescence rise kinetics OJIP were measured with a Handy PEA instrument (Plant Efficiency Analyser, Hansatech Instruments Ltd., King’s Lynn, UK). Samples were always kept in darkness for 0.5 h before the measurements and were illuminated with continuous red light (650 nm peak wavelength, 3,500 µmol photons m^−2^ s^−1^ maximum light intensity). The experiment was repeated three times with at least 15 repetitions. For *C. reinhardtii*, 1 ml of cells in Buffer A with 0.65 A_750_ were treated with 1% DMSO (mock), GT (10, 50, and 100 μM) and 1 μM DCMU for 2.5 h under 100 µmol m^−2^ s^−1^ white light at 25°C. The samples were collected by centrifugation and resuspended in 20 µl Buffer A. After 0.5 h dark-adaptation, 20 µl suspensions were filtered onto glass microfiber filter (diameter 25 mm, GF/C, Whatman), and then positioned immediately above the PEA sensor head by a leaf clip to obtained the fluorescence data. For thylakoids of spinach (*Spinacea oleracea*), thylakoids were isolated according to the method of Chen et al. (2008). Before the fluorescence OJIP curves measurements, 1% DMSO (mock), GT (50, 100, 200, and 400 μM) and 1 μM DCMU were added to thylakoid suspensions with 100 μg Chl ml^−1^ and incubated for 0.5 h in complete darkness at 25 °C.

The fluorescence rise kinetics OJIP curves were analyzed by the JIP-test based on the model of “Theory of Energy Fluxes in Biomembranes” (Strasser et al., 2004). The JIP-test defines the specific (per reaction center, RC) and the phenomenological (per excited cross-section, CS) energy fluxes of the absorbed light by the antenna pigments (ABS), the maximum energy trapping (TR_0_), the electron transport beyond Q_A_ **^-^** (ET_0_) and dissipation (DI_0_). Various JIP-test parameters used in this study are listed in **Table 1**.

The MR_820_ signal measurements of *C. reinhardtii* cells were performed using a Multifunctional-PEA fluorometer (Hansatech Instruments Ltd., King’s Lynn, UK). Further technical details were described in a reference by Gao et al. (2018). The raw data were transferred to the computer and the numerical processing of the MR_820_ signals were carried out by the in-house software M-PEA data Analyzer v.5.1.

### Modeling of GT in the Q_B_-Binding Site

Based on the assumption that the Dl protein is an equivalent of the L-subunit, the coordinates of the L-subunit of purple photosynthetic bacterial *Rhodopseudomonas viridis* obtained from Protein Data Bank (PDB entry 1PRC) were used as the templates for the Dl protein. The amino acid sequence information of target protein Dl of *C. reinhardtii* (Reference Sequence: NP_958413.1) and *S. oleracea* (Reference Sequence: NP_054912.1) was obtained from NCBI. Docking was performed with DS-CDocker implemented in Discovery Studio (version 3.5, BIOVIA, America). The modeling started from the crystal structure alignment of complexes of the bacterial *Rps. viridis* RC with atrazine (Lancaster and Michel, 1999; PDB entry 5PRC) and the crystal structure alignment of *S. oleracea* D1 protein (PDB entry 3JCU). The atrazine binding environment in the bacterial RC or atrazine, DCMU and toxin GT binding environment in *S. oleracea* D1 was further refined by molecular dynamics simulations. The structures of three ligands were constructed using ChemBioDraw Ultra 14.0 software (CambridgeSoft, America). The ligand structures were energetically minimized using MM2 energy minimizations in Chem3D Pro 14.0 (CambridgeSoft, America). All bound water and ligands were eliminated from the protein, and the polar hydrogens were added to the proteins in the processes of the above energy minimization and molecular refinement.

## Results and Discussion

### GT Caused Leaf Lesion of Various Monocotyledonous and Dicotyledonous Plants

**Figure 1:**
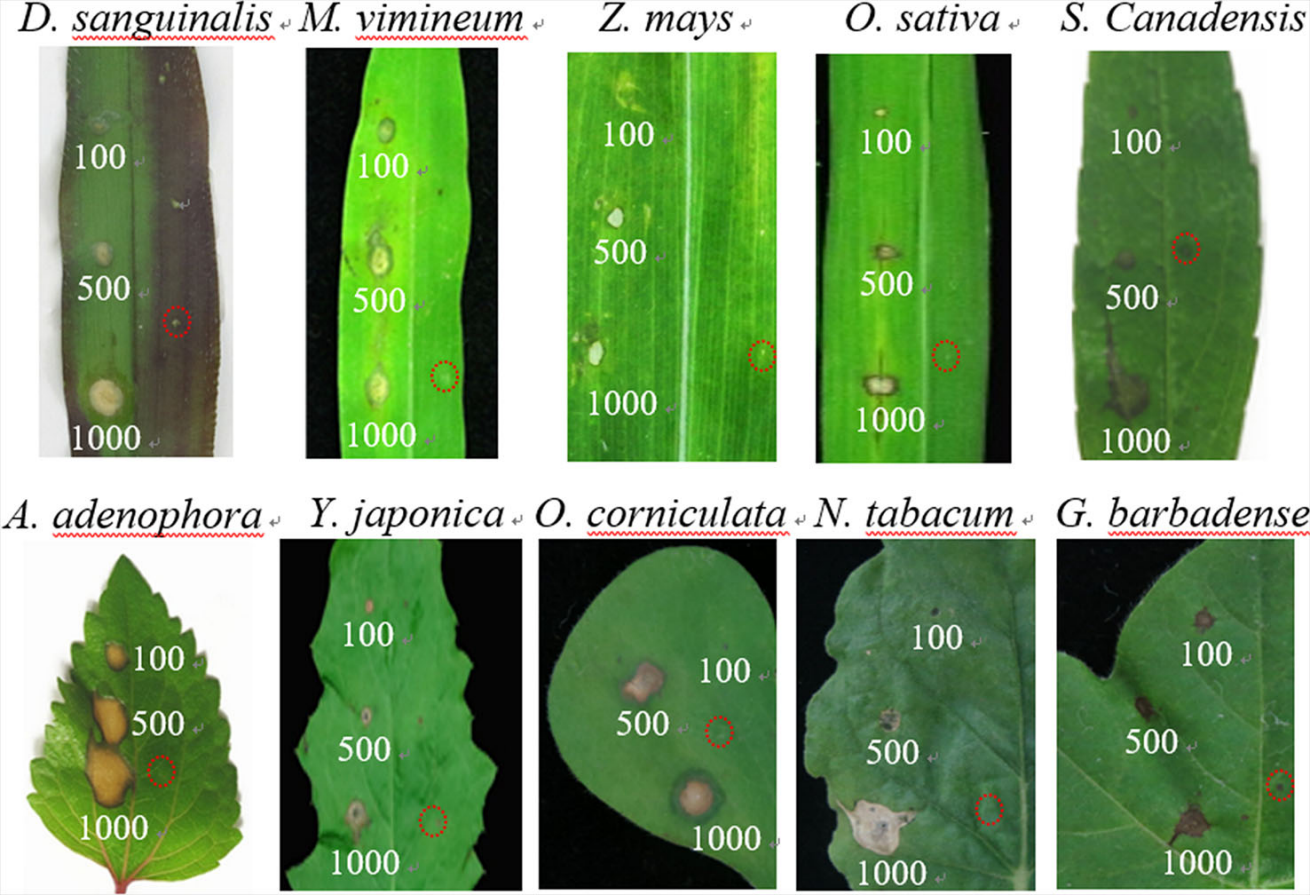
Disease development of the detached-leaves with Gliotoxin (GT). Leaves from 10 different plant species were treated without (1% DMSO as control, red circles on the right side) or with GT at various concentrations (100, 500, and 1,000 µM). Lesion photographs were taken at 96 h. Lesion diameter and pathogenicity level were analyzed in **Table 2**.

It was proved that GT possesses growth inhibiting bioactivity (Wright, 1951; Furuta et al., 1984; Haraguchi et al., 1992; Haraguchi et al., 1996; Haraguchi et al., 1997). To further examine the phytotoxicity of GT to different plants, the leaf lesion formation was monitored. As shown in **Figure 1**, the ratio of lesions in the leaf blades of ten plant species exhibited a concentration-dependent increase after 96 h treatment with 100, 500, and 1,000 µM GT. The white leaf spot was observed in GT-treated five plants including *Digitaria sanguinalis*, *Microstegium vimineum*, *Zea mays*, *Oryza sativa,* and *Nicotiana tabacum*. GT caused brown leaf spot in another five plants including *Solidago Canadensis, Ageratina adenophora, Youngia japonica, Oxalis corniculata, and Gossypium barbadense*. In the case of 1% DMSO treatment (mock), no visible damage was found in the leaf blades in these ten plants. Such chlorosis or necrosis symptoms indicate that GT led to chlorophyll breakup and cell death.

To further access pathogenicity level of GT, these ten plant species were classified into three categories according to the diameter of leaf lesions (**Table 2**). At 100 µM GT, *A. adenophora* leaves showed highly susceptible, where the diameter of the developed necrotic lesion in leaf blades was more than 3.0 mm. For lower susceptible *D. sanguinalis*, their leaves formed chlorotic lesions of less than 1.0 mm in diameter. The left eight species belonged to moderately susceptible category with leaf lesions between 1.0 and 3.0 mm in diameter, including monocotyledonous (e.g., *M. vimineum*, *Z. mays,* and *O. sativa*), and dicotyledonous plants (e.g., *S. canadensis*, *Y. japonica*, *O. corniculata*, *N. tabacum* and *G. barbadense*). Based on such standard, *D. sanguinalis*, *O. sativa*, *G. barbadense* leaves still show moderate pathogenicity level at higher concentrations of 500 even 1000 µM GT. Obviously, GT has good phytotoxicity to various monocotyledonous and dicotyledonous plants, leading to leaf lesion formation. It is also suggested that GT can damage photosynthetic tissues because chlorotic or necrotic lesions are evidence of chlorophyll destruction and cell death.

**Table 2:** Phytotoxicity of Gliotoxin (GT) to various plants^a^.

Family	Plant species	GT concentration (µM)	Lesion diameter (mm)	Pathogenicity level^b^
Gramineae	*Digitaria sanguinalis*	100	0.61 ± 0.15	+
		500	2.24 ± 0.16	++
		1,000	2.59 ± 0.09	++
	*Microstegium vimineum*	100	2.49 ± 0.13	++
		500	3.06 ± 0.10	+++
		1,000	4.23 ± 0.66	+++
	*Zea mays Linn*	100	2.10 ± 0.16	++
		500	3.60 ± 0.83	+++
		1,000	5.49 ± 0.29	+++
	*Oryza sativa*	100	1.29 ± 0.09	++
		500	2.09 ± 0.07	++
		1,000	2.10 ± 0.07	++
Compositae	*Solidago Canadensis*	100	2.10 ± 0.67	++
		500	3.19 ± 1.51	+++
		1,000	3.60 ± 0.69	+++
	*Ageratina adenophora*	100	3.88 ± 0.34	+++
		500	5.94 ± 1.17	+++
		1,000	7.25 ± 1.69	+++
	*Youngia japonica*	100	1.68 ± 0.13	++
		500	3.04 ± 0.21	+++
		1,000	3.78 ± 0.22	+++
Oxalidaceae	*Oxalis corniculata L*	100	2.55 ± 0.08	++
		500	3.79 ± 0.24	+++
		1,000	4.20 ± 0.29	+++
Solanaceae	*Nicotiana tabacum L.*	100	1.58 ± 0.10	++
		500	2.87 ± 0.60	++
		1,000	4.84 ± 2.05	+++
Malvaceae	*Gossypium barbadense*	100	1.63 ± 0.18	++
		500	1.96 ± 0.11	++
		1,000	2.61 ± 0.17	++

^a^The detached-intact leaves from different plant species are rinsed with sterilized water, subsequently dried and placed in Petri dishes with wet filter paper. The leaves were lightly punctured using a needle from the leaf margin on the abaxial side. Ten microliter of GT solution was dripped onto the punctured wound. All Petri dishes were placed in the growth chamber for 96 h at 25°C under around 200 µmol m^−2^ s^−1^ white light (day/night, 12 h/12 h). Diameter of leaf lesion was measured with calipers. Each value is the average of three independent experiments. ^b^ +, ++, +++ denotes leaf lesion diameter 0 to <1.0 mm, 1.0 to <3.0 mm, and ≥3.0 mm, respectively.

### GT Decreased the Oxygen Evolution Rate of *C. reinhardtii*

**Figure 2:**
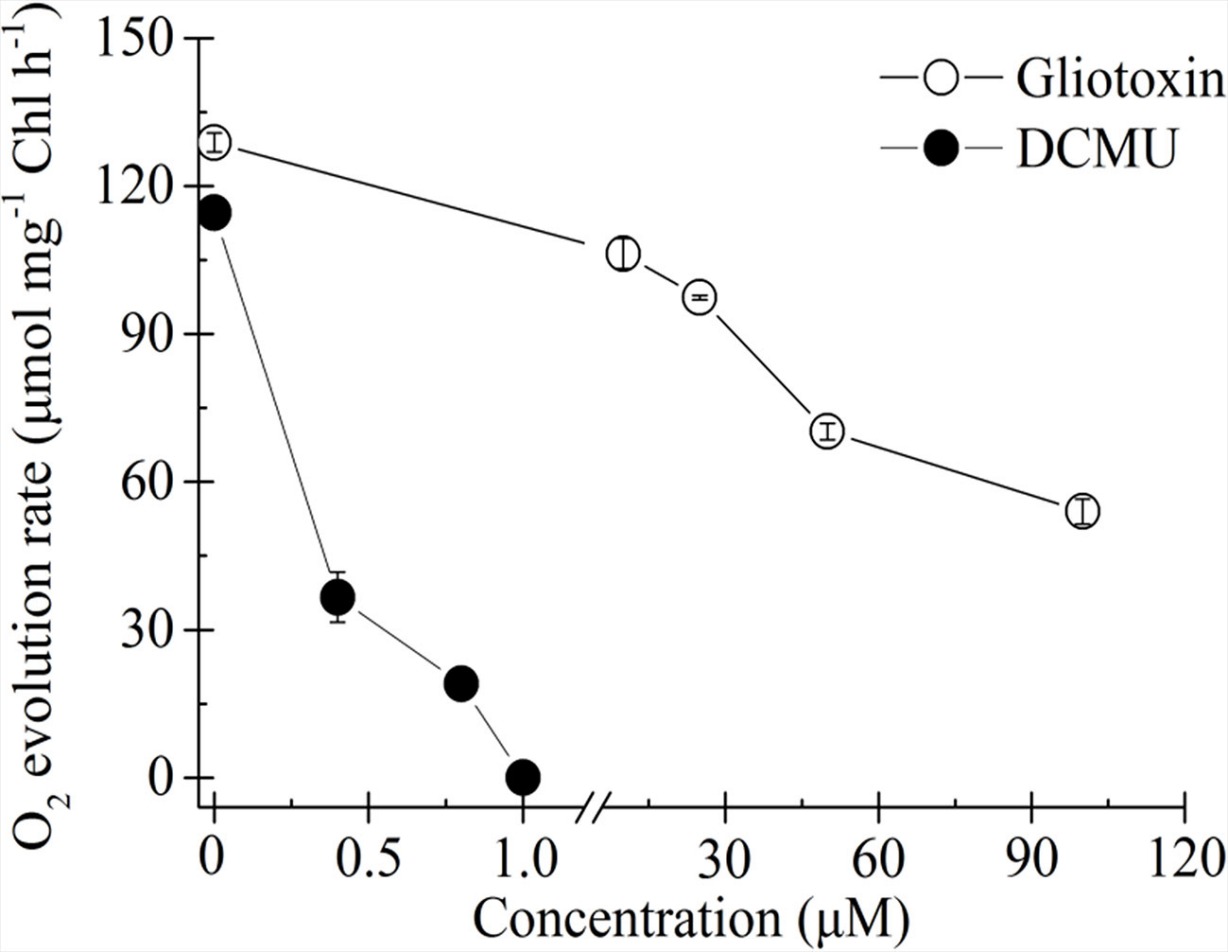
Effect of Gliotoxin (GT) and DCMU on the rate of O_2_ evolution of *C. reinhardtii* cells. H_2_O and p-phenylenediamine is the electron donor and acceptor, respectively. Data shown are mean values ± SE of 3 times independent measurements.

Influence of GT and DCMU on the rate of O_2_ evolution of *C. reinhardtii* cells is shown in **Figure 2**. DCMU inhibits O_2_ evolution much faster and at lower concentrations compared with GT. Almost 100% decrease in O_2_ evolution was observed in the case of 1 µM DCMU treatment. GT also caused a negative concentration-dependent effect on O_2_ evolution. More than 58% decrease in the O_2_ evolution rate had occurred when *C. reinhardtii* cells were exposed to 100 µM GT comparable to mock-treatment. The *I*_50_ (the concentration producing 50% inhibition) value of GT for the inhibition of O_2_ evolution *in vivo* was calculated to be around 60 µM. Clearly, GT is a weaker photosynthetic inhibitor relative to DCMU as an excellent photosynthetic inhibiting herbicide.

### GT Inhibited Photosynthetic Activity of *C. reinhardtii*

**Figure 3:**
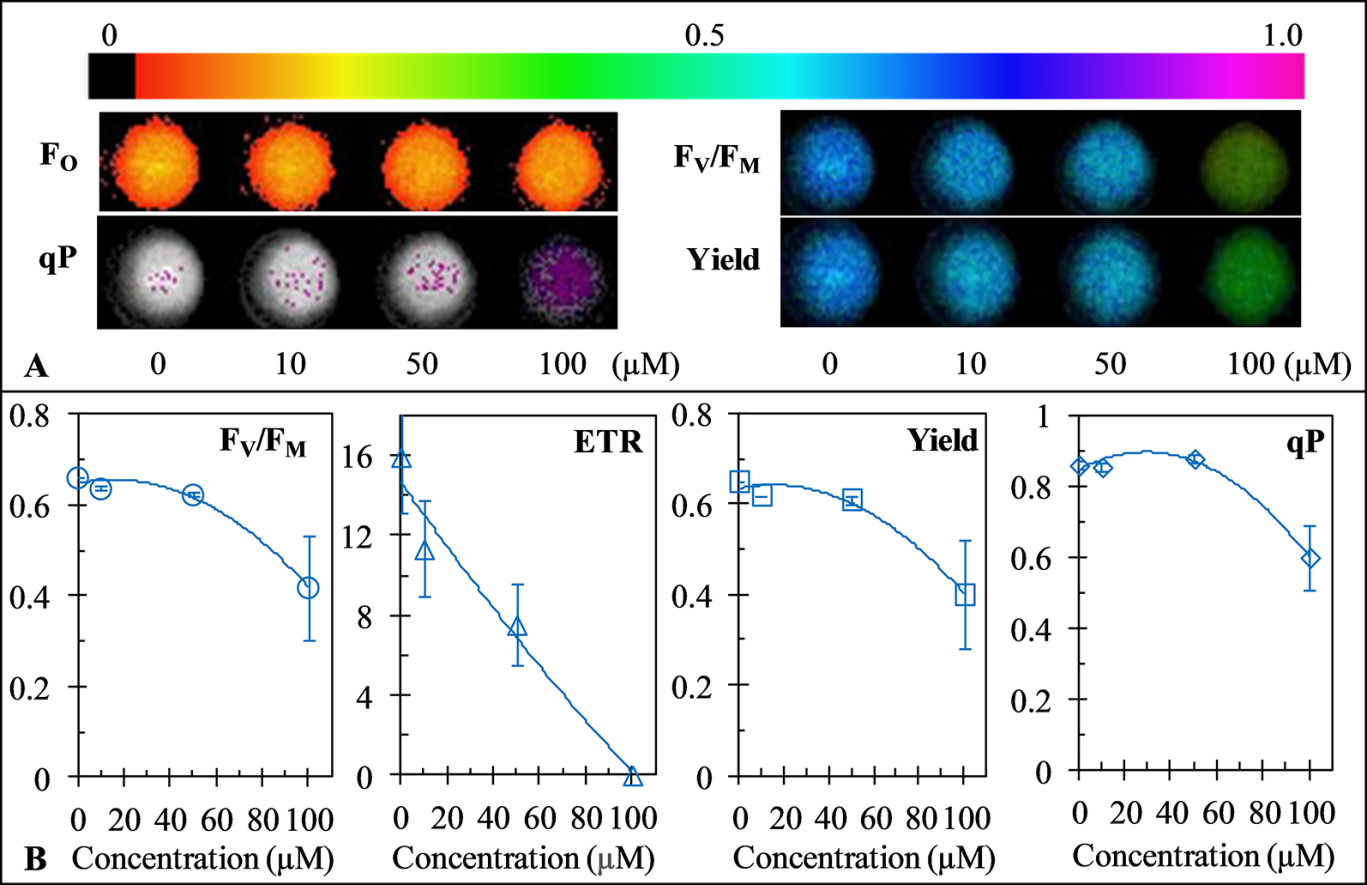
Effect of Gliotoxin (GT) on color fluorescence imaging **(A)** the value of the maximum quantum yield of PSII (F_V_/F_M_), electron transport rate (ETR), Yield and qP **(B)** of *C. reinhardtii* cells. Fluorescence images were indicated by color code in the order of black (0) through red, orange, yellow, green, blue, violet to purple (1). The number codes above images are marked from 0 to 1, showing the changes. Each value is the average ± SE of three independent experiments.

Chlorophyll fluorescence is an indicator of plant photosynthetic activity. To test the effect of GT on photosynthesis of *C. reinhardtii*, the Imaging-PAM chlorophyll fluorometer was used to monitor the change of GT-induced fluorescence image parameters. As in **Figure 3A**, the representative color-coded images of four parameters, F_O_ (when all PSII reaction centers are open after dark adaptation), F_V_/F_M_ (the maximal PSII quantum yield), qP (the coefficient of photochemical quenching), and Yield (the effective PSII quantum yield), are shown after *C. reinhardtii* cells were treated with different concentrations of GT. It is observed that images of F_O_ kept relatively stable in the presence of GT, images of F_V_/F_M_ and Yield as well as qP also did not been affected in the case of 10 and 50 µM GT treatment. At 100 µM GT-treated *C. reinhardtii* cells, images of F_V_/F_M_ and Yield faded from blue for mock to green, images of qP became purple colors from white colors. The results are strongly supported by the values of fluorescence parameters F_V_/F_M_, Yield and qP (**Figure 3B**). About 36% and 38% decrease in F_V_/F_M_ and Yield, respectively, was observed relative to mock-treatment after 100 µM GT treatment, suggesting high concentration of GT declined the quantum efficiency of light energy transfer in PSII. The value of qP was 0.86 for mock-treatment, and was 0.60 for 100 µM GT-treatment. qP denotes the proportion of excitons captured by open traps and being concerted to chemical energy in the PSII reaction center (Krause and Weis, 1991). In addition, the parameter ETR, expressing the apparent rate of photosynthetic electron transport, exhibited a rapid concentration-dependent decrease by increasing of GT concentration (**Figure 3B**). After *C. Reinhardtii* cells were incubated by 10, 50, and 100 µM GT, the mean of ETR was declined by around 29%, 52.%, and 100% by comparison with mock-treatment, respectively. The *I*_50_ value for ETR is about 50 µM, which is closed to the *I*_50_ value for O_2_ evolution rate. An approximately linear lower in ETR and O_2_ evolution rate indicates that the inhibition of PSII electron transport should be the important action site of GT on photosynthetic apparatus. Considering above results, it is concluded that GT can affect photosynthesis of *C. Reinhardtii* mainly due to inhibiting PSII electron transport.

### Action Sites of GT on Photosystem II and Photosystem I of *C. reinhardtii*

**Figure 4:**
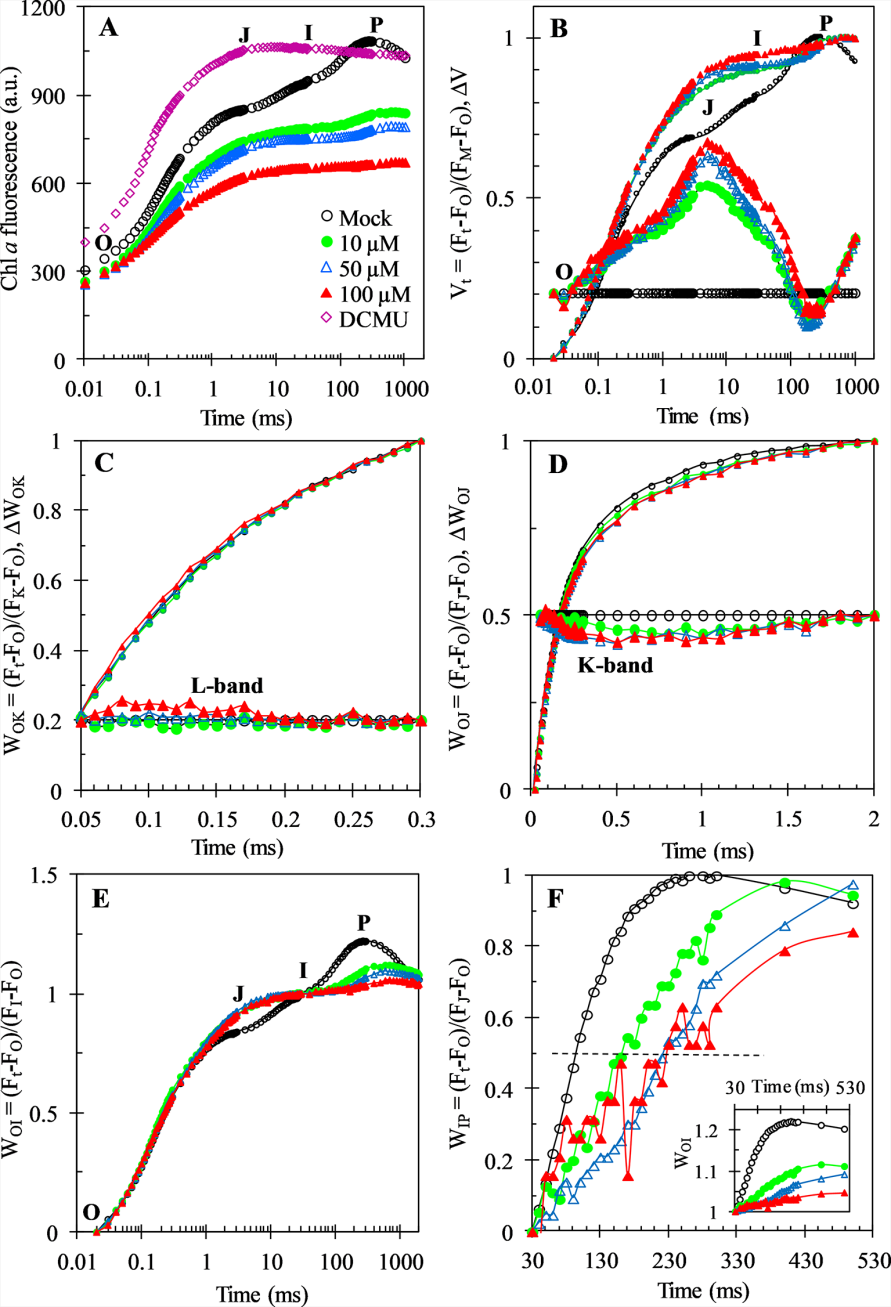
Chl *a* fluorescence rise kinetics of *C. reinhardtii* cells treated with 1% DMSO (mock), DCMU (1 μM), and Gliotoxin (GT) at the indicated concentrations. **(A)** Raw fluorescence rise kinetics. **(B)** Fluorescence rise kinetics normalized by F_O_ and F_M_ as V_t_ = (F_t_ − F_O_)/(F_M_−F_O_) (top), and ΔV_t_ = Vt_(treated)_ − V_t(control)_ (bottom). **(C)** Fluorescence rise kinetics normalized by F_O_ and F_K_ as W_OK_ = (F_t_ − F_O_)/(F_K_ − F_O_) (top), and the difference kinetics ΔW_OK_ = W_OK(treated)_ − W_OK(control)_ (bottom). **(D)** Fluorescence rise kinetics normalized by F_O_ and F_J_ as W_OJ_ = (F_t_ − F_O_)/(F_J_ − F_O_) (top), and the difference kinetics ΔW_OJ_ = W_OJ(treated)_ − W_OJ(control)_ (bottom). **(E)** Fluorescence rise kinetics normalized by F_O_ and F_I_ as W_OI_ = (F_t_ − F_O_)/(F_I_ − F_O_). **(F)** Fluorescence rise kinetics normalized by F_I_ and F_P_ as W_IP_ = (F_t_ − F_I_)/(F_P_ − F_I_) and W_OI_ (≥1) in the insert, the half-times are shown by the crossing of the curves with the horizontal dashed line drawn at W_IP_ = 0.5 (half rise). Each curve is the average of 30 measurements.

In the last two decades, fast chlorophyll a fluorescence rise kinetics OJIP and JIP-test analysis has been widely used to probe the structure, conformation and function of the photosynthetic apparatus (Strasser et al., 1995; Strasser et al., 2004). To further investigate the precise action sites of GT on photosynthesis, the fluorescence rise kinetics OJIP of *C. reinhardtii* cells were measured after GT treatment for 3 h with different concentrations (**Figure 4**).

As shown in **Figure 4A**, the fluorescence rise OJIP curve of mock-treatment is a typical polyphasic O-J-I-P shape. GT and DCMU treatment led to a distinct change of the fluorescence rise OJIP curve of *C. reinhardtii* cells. After 1 μM DCMU treatment, the biggest change of fluorescence rise OJIP curve is that the J-step increased quickly equal to the P level (F_M_). A rapid rise of the level of J-step is a result of the large accumulation of Q_A_^-^ in PSII RCs, which attributes to the interruption of the electron flow from Q_A_ to Q_B_ (Strasser and Govindjee, 1992; Strasser et al., 2004). For GT, it is observed that the variable Chl fluorescence intensity (Ft) and F_M_ decreased significantly, and the I- and P-steps disappeared gradually by increasing treatment concentration. A decrease in F_M_ might be relative to the quenching of ﬂuorescence, which is resulted from the presence of an oxidized plastoquinone pool or to the damage of the structure and function of PSII antennae (Tóth et al., 2005).

For investigation of the detailed effect of GT on Chl *a* fluorescence rise kinetics OJIP properties, the fluorescence curves were double normalized by F_O_ and F_M_, and presented as relative variable fluorescence V_t_ (top) and ΔV_t_ = V_t(treated)_ − V_t(control)_ (bottom) versus logarithmic time scale (**Figure 4B**, the “control” is the mock-treated samples). This allows to find richer information that usually were hidden in the actual fluorescence rise kinetics curve. The data from the V_t_ and ΔV_t_ show that the most major effect of GT on the fluorescence rise kinetics is a rapid increase of the J-peak. This is equivalent to the DCMU behavior. To analyze GT-treated cells for events, reflected in the OK, OJ, OI, and IP phase, other normalizations of the fluorescence rise kinetics were also conducted (**Figures 4C-F**). In **Figure 4C**, the fluorescence rise kinetics data were double normalized by F_O_ and F_K_ as W_OK_ (top) and plotted with ΔW_OK_ = W_OK(treated)_ − W_OK(control)_ (bottom) to show L-band. The L-band is an indicator of the energetic connectivity or grouping of the PSII units, being higher when connectivity or grouping probability is lower (Strasser et al., 2004). Our data reveal that L-band is low sensitive to different concentration of GT. In **Figure 4D**, the fluorescence rise kinetics normalized by F_O_ and F_J_ as W_OJ_ in the linear time scale from 10 μs to 2 ms is presented. No clear effect on the OJ phase was observed after *C. reinhardtii* cells were incubated by GT. Based on the ΔW_OJ_ = W_OJ(treated)_ − W_OJ(control)_, it is seen that GT just caused a very slight negative influence on the K-band (**Figure 4D**). The OJ phase is largely driven by primary photochemistry, the JP phase is dominated by the biochemical reaction (Strasser et al., 2004). So, it seems reasonable that the mainly influence of GT is on the biochemical reaction after Q_A_ not the primary photochemical reaction. **Figure 4E** shows that the fluorescence rise kinetics were double normalized by F_O_ and F_I_ as W_OI_. The J-peak of GT-treated curves exhibited a significant increase compared with that of mock. At the same time, the W_OI_ (only the part ≥ 1 is shown), in the linear 30–530 ms time range, was also plotted in the insert in **Figure 4F**. The I-step reflects the kinetic bottleneck of the electron chain between PQH_2_ and cytochrome (cyt) b_6_f (Strasser et al., 2010). The IP phase is related to electron flow through PSI and inactive ferredoxin-NADP^+^-reductase (FNR) at the acceptor side of PSI (Schansker et al., 2005). It reflects the electron flow from PQ pool to the end electron acceptors at the PSI acceptor side. For each W_OI_ curve, the maximal amplitude of the fluorescence rise from I- to P-step reflects the size of the pool of the end electron acceptors at PSI acceptor side (Yusuf et al., 2010; Chen et al., 2016). It is demonstrated that GT resulted in a decrease of this pool size since the W_OI_ (≥ 1) curves of GT-treated samples have smaller IP amplitude compared to mock. To further assess the effect of GT on the IP phase, the fluorescence data were normalized by F_I_ and F_P_, as W_IP_ = (F_t_ – F_I_)/(F_P_ – F_I_), and plotted in a linear time scale from 30 to 530 ms (**Figure 4F**). Yusuf et al. (2010) suggested that the reduction rate of PSI end electron acceptors’ pool in different treatments can be estimated by the half-time, which is the time point at W_IP_ = 0.5 (half rise of the curves). A bigger (or less) value of the half-time means a lower (or higher) conduction rate. Here, it is observed that GT caused a distinct increase of the half times relative to mock. This indicates that GT can decline the rate of the reduction of the end electron acceptors on PSI possibly or/and inactivate FNR.

**Figure 5:**
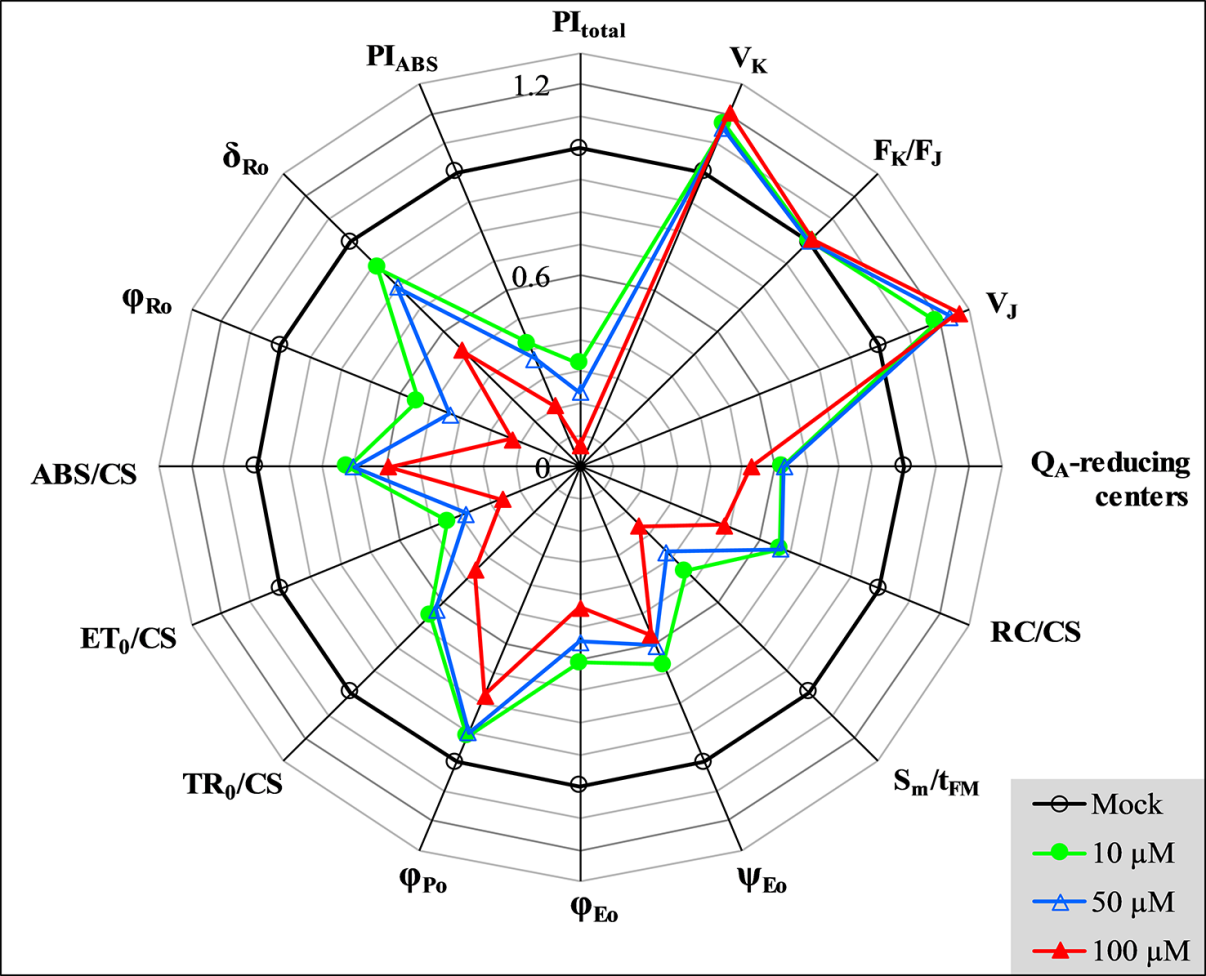
Spider plot presentation of selected parameters derived from JIP-test quantifying PSII behavior of *C. reinhardtii* cells treated with various concentrations of GT. Each parameter is expressed as fraction relatively to the values of the control (mock, back regular circle with value 100% = 1).

In **Figure 5**, several representative JIP-test parameters are presented for further analysis of the behavior of GT-treated *C. reinhardtii* cells. In these parameters, V_K_ (relative variable fluorescence at the K-step) and V_J_ (relative variable fluorescence at the J-step) increased after GT treatment. However, combining with the stable parameter F_K_/F_J_, it’s clear that the increase of V_K_ is caused by the increase of F_J_. Actually, GT has no significant influence on the K-step. It has been suggested that the K-step is a signal of the inactivation of the oxygen-evolving-complex (OEC) (Strasser et al., 2004). Thus, the major impact of GT is a rise of the J-step level, suggesting a large accumulation of Q_A_^-^ occurred in PSII RCs due to inhibition of PSII electron transfer activity. In fact, it is seen that all parameters involved electron transport, ET_0_/CS (electron transport flux per CS), φ_Eo_ (the quantum yield for PSII electron transport), and ψ_Eo_ (the probability that a trapped exciton moves an electron into the electron transport chain beyond Q_A_), show dramatic reduction in the presence of GT. However, GT can’t inhibit entirely PSII electron transport activity *in vivo*. At 100 μM GT, φ_Eo_ and ψ_Eo_ just decreased by around 56% and 43% relative to the mock (**Figure 5**). Since GT blocked PSII electron transfer further than Q_A_, inactivation events of PSII RCs are expected to happen. Data from **Figure 5** show that the number of active PSII RCs per cross-section (RC/CS) decreased quickly after GT treatment. S_m_/t_FM_, expressing the average fraction of open RCs of PSII in the time interval from 0 to t_FM_ (Strasser et al., 2004), is shown. By increasing treatment concentration, an approximately linearly sharply decrease of S_m_/t_FM_ is observed. The S_m_/t_FM_ ratio of GT-treated cells was about 54% (10 μM), 62% (50 μM), and 74% (100 μM) lower than that of mock, respectively. This means that GT caused the faster closure of PSII RCs. The fraction of Q_A_-reducing centers were also calculated according to the reference (Chen et al., 2014), as follow: Q_A_ reducing centers = [(RC/CS)_treatment_/(RC/CS)_control_]. [(ABS/CS)_treatment_/(ABS/CS)_control_]. It is found that the fraction of Q_A_-reducing centers reduced quickly after 3 h treatment of GT with different concentrations (**Figure 5**). The data indicate that GT inactivated indeed the RCs of PSII *in vivo*. In contrast with Q_A_-reducing centers, GT treatment increased the fraction of non-Q_A_-reducing centers (data not shown). Non-Q_A_-reducing centers, also so-called heat sink centers, are radiators and often are used to protect the system from over excitation and over reduction which would create dangerous ROS (Strasser et al., 2004; Chen et al., 2014).

In addition, the maximum quantum yield of PSII primary photochemistry (φ_Po_) shows a slight decrease after *C. reinhardtii* cells were treated with GT *in vivo*. The value of two parameters, ABS/CS and TR_0_/CS decreased greatly. ABS/CS refers to the total absorption flux per PSII cross-section, and can be taken as a measure for an average antenna size or chlorophyll concentration (Srivastava et al., 1998; Strasser et al., 2004). TR_0_/CS expresses the trapped energy flux per PSII cross-section, reflecting the specific rate of the exciton trapped by open RCs (Strasser et al., 2004). A significant decrease of ABS/CS and TR_0_/CS indicates that GT not only lowered the chlorophyll concentration, but damaged the conformation of the antenna pigment assemblies and reduced the efficiency of light energy transfer between antenna pigment molecules and from those to the PSII RCs. This might be also a possibility for decreasing of the variable Chl fluorescence intensity (F_t_) and the F_M_ value. With respect to the performance index PI_ABS_, the parameter is a product of the three independent parameters φ_Po_, ψ_Eo,_ and γ_RC_. Here, γ_RC_ is the fraction of RC chlorophyll in relation to total chlorophyll (Strasser et al., 2004; **Table 1**). So, PI_ABS_ is extremely sensitive to different stresses, expressing the overall photosynthetic activity of PSII (Strasser et al., 2004). When cells were exposed to 10, 50, and 100 μM GT, the value of PI_ABS_ decreased by 58%, 63%, and 79% compared to the mock, respectively. It is proved that GT can strongly inhibit PSII photosynthetic activity of *C. reinhardtii*. Moreover, PI_ABS_ is much higher sensitive to GT treatment than these three parameters (φ_Po_, ψ_Eo_, and γ_RC_) that contribute to this index and even PSII O_2_ evolution rate (**Figures 2** and **5**). This might be interpreted by the reason that the inhibition of PSII electron transfer activity is the dominant factor not the only one for the decrease of the overall photosynthetic activity of PSII after GT treatment.

After *C. reinhardtii* cells were treated with various concentrations of GT, both φ_Ro_ and δ_Ro_ show a significant decrease. The value of φ_Ro_ reduced to 54% (10 μM), 44% (50 μM), and 23% (100 μM) of the mock, respectively (**Figure 5**). For 10 μM and 50 μM GT treated cells, δ_Ro_ only lowered 12% and 20%. Under 100 μM GT treatment, δ_Ro_ has 48% distinct decrease. φ_Ro_ is the product of φ_Po_, ψ_Eo_, and δ_Ro_, expressing that the quantum yield for the reduction of the end electron acceptors at the PSI acceptor side (Strasser et al., 2010). Here, δ_Ro_ is given as δ_Ro_ = RE_0_/ET_0_ = (1−V_I_)/(1−V_J_). δ_Ro_ is used as the probability that an electron is transported from the reduced intersystem electron acceptors to final electron acceptors of PSI (Strasser et al., 2010). A significant decrease of φ_Ro_ and δ_Ro_ suggests that GT inhibits the reduction of the end acceptors at the PSI electron acceptor side. This is well in agreement with that GT-treated cells have a smaller IP amplitude during Chl fluorescence transient (**Figure 4F**). Ceppi et al. (2012) suggested that the IP amplitude is a semiquantitative indicator for relative changes in the PSI content. A smaller IP amplitude is related to a loss of PSI content (Oukarroum et al., 2009; Ceppi et al., 2012). A decrease of PSI content is due to increased level of PSI produced oxygen radicals (Mittler, 2002; Oukarroum et al., 2009). A block of electron flow on the acceptor side of PSI will divert electrons from the PSI acceptor side, that normally goes to carbon fixation path, to reduce O_2_ generating ROS (Chen et al., 2012). Another possible explanation for the decrease in IP amplitude would seem to be a loss of cyt b_6_/f complexes. Because the loss of cyt b_6_/f complexes could make the rate limitation posed by the re-oxidation of PQH_2_ stronger and as a consequence shift the I-step up. This could also lead to a smaller IP amplitude not directly relative to a loss of PSI (Ceppi et al., 2012). The performance index PI_total_ incorporates two parameters PI_ABS_ and δ_Ro_, reflecting the whole photosynthetic activity of two photosystems (Strasser et al., 2010). Here, PI_total_ is the most sensitive JIP-test parameter. After cells were treated with 10, 50, and 100 μM GT, PI_total_ declined to about 33%, 24%, and 7% of mock.

**Figure 6:**
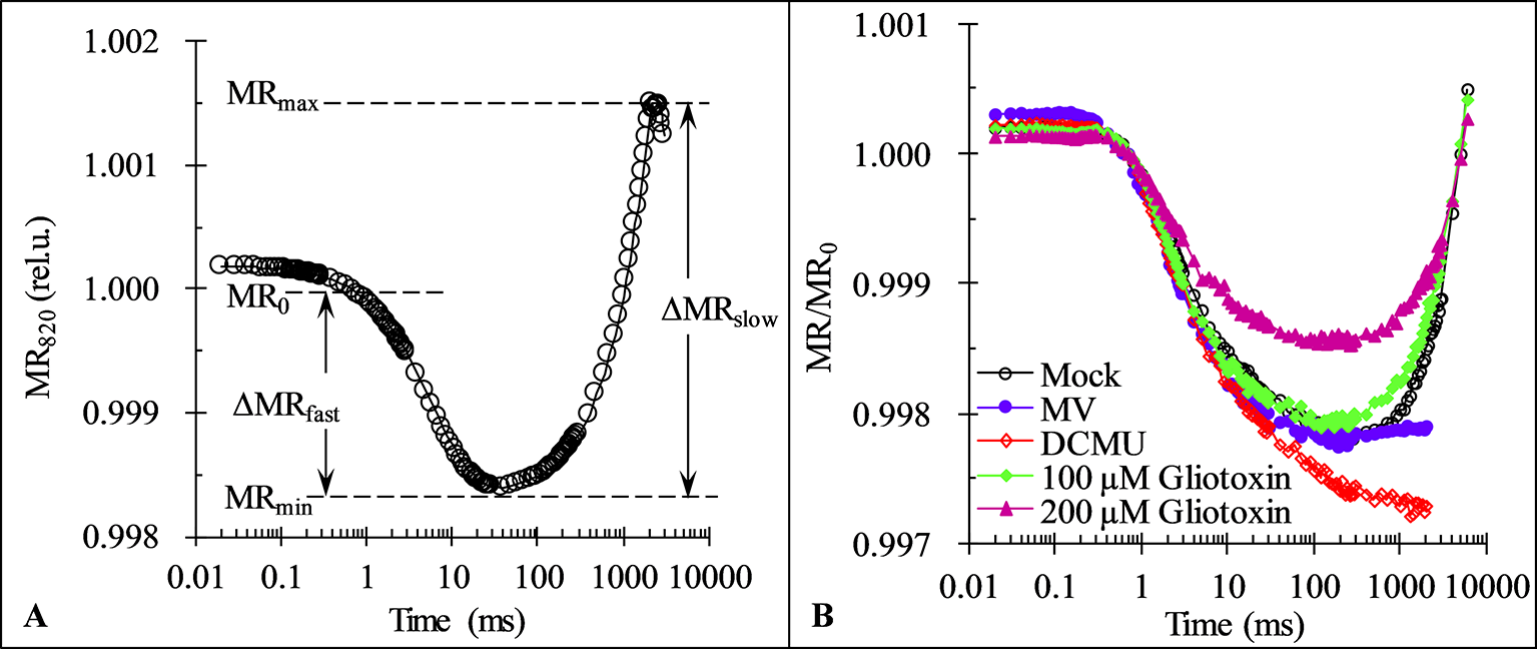
Effect of Gliotoxin (GT) on the kinetics of modulated reflection at 820 nm (MR). **(A)** A graphical definition of the characteristic parameters of the MR kinetics. Here, MR_0_ is the value at the onset of the actinic illumination (taken at 0.7 ms, the first reliable MR measurement), MR_min_ is the minimal signal reached during the fast phase between 0.7 ms and 10–200 ms, MR_max_ is the maximal signal reached by the end of the slow phase (usually taken at 1 to 2 s). **(B)** The MR induction curves of *C. reinhardtii* cells treated with 1% DMSO (mock), MV (200 µM), DCMU (1 μM), and GT (100, 200 µM). The plotted values are expresses by the MR/MR_0_ ratio. Each curve is the average of 30 measurements.

Fluorescence rise from I- to P-step lasts normally from around 30 to 200 ms and is shown to parallel the re-reduction of plastocyanin (PC^+^) and PSI reaction center (P_700_^+^) (Strasser et al., 2010). Here, in order to further confirm the effect of GT on PSI, the modulated reflection at 820 nm (MR) of *C. reinhardtii* cells was determined. As shown in **Figure 6A**, a typical MR signal curve exhibits two phases: a fast decrease phase between MR_0_ (about 0.7 ms) and MR_min_ (about 10–200 ms), and a slow increase phase between MR_min_ (about 10–200 ms) and MR_max_ (about 1–2 s). The fast phase corresponds to the accumulation of PC^+^ and P_700_^+^. The transitory steady state of MR kinetics at the end of the fast phase, MR_min_, appears due to the accumulation of PSII initiates electron transfer to PC^+^ and P_700_^+^ just compensates the further oxidation of PC and P_700_ by PSI activity. In other words, at the MR_min_ point the non-cycle electron flow through PSII and PSI achieved the balance level. Once the reduction rate overcomes the oxidation rate, the slow MR phase comes out. The slow phase corresponds to the net re-reduction of PC^+^ and P_700_^+^ by the intersystem electron carriers (Strasser et al., 2010; Goltsev et al., 2012). At 100 μM GT, 1 μM DCMU, and 200 µM MV, a similar the fast phase of MR/MR_0_ kinetics of cells was observed compared to mock, reflecting no effect on the capability of P_700_ to get oxidized. For MV as a PSI herbicide, it gets electrons from PSI electron transport chain at the nearly same rate as PSII is pumping them to the PSI (Schansker et al., 2005). Therefore, the MR signal of MV-treated cells remained the steady same level as the MR_min_ after the end of the fast MR phase (**Figure 6B**). However, the slow MR phase of DCMU-treated cells was losing, suggesting the complete disconnection of two photosystems (**Figure 6B**). In the presence of DCMU, the fast MR phase continnued to go down, revealing a further more oxidation of PC and P_700_. This is because that DCMU can prevent entirely PSII electron flow from reaching the PC and P_700_ and oxidating them (Schansker et al., 2003). A smaller slow MR phase reveals a lower rate of the net re-reduction of PC^+^ and P_700_^+^ in the case of GT with 100 and 200 μM. Obviously, unlike DCMU, GT could not completely inhibt electron flow from PQ at PSII acceptor side to PC^+^ and P_700_^+^ since the slow MR phase did not disappear. At higher concentration of 200 μM GT, a pronounced decrease of the fast MR phase was also observed, indicating the capability of P_700_ to get oxidized was markedly decreased (**Figure 6B**). Considering the decrease in the IP amplititude of OJIP curve, it is suggested that GT cause a loss of PSI active contents and disconnection of two photosystems.

On the basis of above analysis of *C. reinhardtii* cells *in vivo*, it is concluded that the major action site of GT is the acceptor side of PSII, blocking electron transfer further than Q_A_ and then inactivating PSII RCs. In addition, GT can also destroy the antenna pigment assemblies and damage the reduction of the end acceptors at the PSI acceptor side.

### Effects of GT on Photosystem II and Photosystem I of Spinach Thylakoids

**Figure 7:**
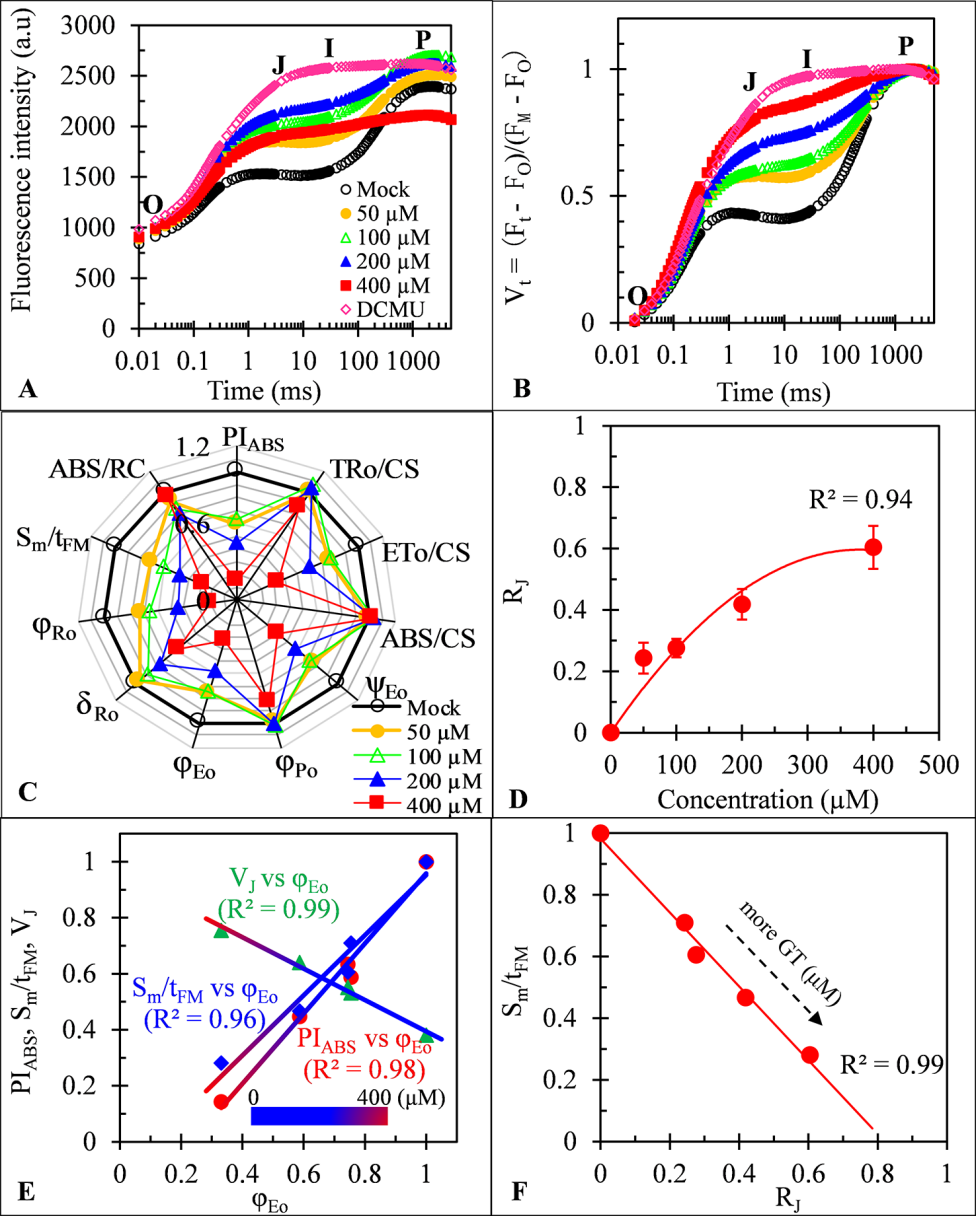
Effect of Gliotoxin (GT) on spinach thylakoids. **(A)** Raw fluorescence rise kinetics of thylakoids treated with 1% DMSO (mock), DCMU (1 μM), and GT at the indicated concentrations for 0.5 h. **(B)** Fluorescence rise kinetics normalized by F_O_ and F_M_ as V_t_ = (F_t_ − F_O_)/(F_M_−F_O_). **(C)** Radar plot presenting the JIP-test parameters from thylakoids with different concentrations of GT. **(D)** The concentration-dependent change of R_J_. The parameter R_J_ reflects the number of PSII RCs with their Q_B_ site filled by PSII inhibitors (here is GT). **(E)** Analysis of the correlation for V_J_, PI_ABS_, and S_m_/t_FM_ versus φ_Eo_ of spinach thylakoids treated with GT at different concentration (mock, 50, 100, 200, and 400 μM). **(F)** Analysis of the linear relationship between S_m_/t_FM_ and R_J_ after spinach thylakoids were treated with GT. Each value is the average of 30 measurements.

To more in-depth investigate the direct action sites of GT on two photosystems, Chl fluorescence rise kinetics OJIP of spinach thylakoids were measured and analyzed. As *C. reinhardtii* cells *in vivo*, the best biggest change of the fluorescence rise OJIP curves of GT-treated thylakoids is a significant rise of the J-step level (**Figures 7A, B**). A similar effect of GT is also found on JIP-test parameters including φ_Po_, ET_0_/CS, φ_Eo_, ψ_Eo_, S_m_/t_FM_, PI_ABS_, φ_Ro_, and δ_Ro_ (**Figures 5** and **7C**). Moreover, the J-step level (V_J_), PI_ABS_, and S_m_/t_FM_ are linearly related to φ_Eo_ by increasing of GT concentration (**Figure 7E**). Such results further prove that GT mainly inhibits the PSII overall activity by inactivating PSII RCs due to interruption of PSII electron transfer beyond Q_A_ at the acceptor side. Concerning the values of δ_Ro_, the inhibition of the reduction of the end acceptors at the PSI electron acceptor side is another action site of GT at high concentration above 100 μM. The conclusion is consistent with the previous analysis of the MR_820_ kinetics. However, unlike *C. reinhardtii* cells, evidence from ABS/CS and TR_0_/CS shows that no remarkable direct influence on the chlorophyll concentration and antenna pigment assemblies of PSII was observed in GT-treated spinach thylakoids (**Figure 7C**). Another expression ABS/RC, being taken a calculated average amount of chlorophyll which channels excitation energy into RC (Srivastava et al., 1998), also did not respond to different concentrations of GT treatment. Consequently, it is assumed that the damage of PSII antenna pigment assemblies in GT-treated *C. reinhardtii* cells *in vivo* might be an indirect effect of ROS production attributed to inactivation of PSII RCs and inhibition of the reduction of the end acceptors at the PSI acceptor side. It is further supported by the evidence from SDS-PAGE of thylakoid membrane proteins. After 50, 100, 200, and 400 μM GT treatment, the content of major PSI and PSII polypeptides, including PSI core peptide PsaA/B, D1/D2 dimer, PSII core antenna chl-binding protein (CP47, CP43, and CP29) and OEC 33 kD, did not show remarkable differences compared with the mock and DCMU treatment (see **Figure S1**). It is indicated that GT does not alter directly thylakoid polypeptide composition.

Previous evidence has tangibly demonstrated that PSII inhibiting herbicides lead to inactivation of PSII RCs for blocking electron flow beyond Q_A_ due to the Q_B_-site of D1 protein occupying by herbicide molecules (Lazár et al., 1998; Oettmeier, 1999). Based on the JIP-test, the parameter R_J_ is derived, which represents the number of PSII RCs with Q_B_-site filled by PSII inhibitor (**Table 1**; Lazár et al., 1998; Chen et al., 2014). The data in **Figure 7D** show that the value of R_J_ increases with the increasing concentration of GT. After spinach thylakoids were incubated with GT for 0.5 h, the amount of PSII RCs with Q_B_-site filled by GT was about 24% (50 μM), 28% (100 μM), 42% (200 μM), and 60% (400 μM), respectively. There is a visible concentration-dependent enhancement of GT bound to PSII RCs. A highly negative correlation between S_m_/t_FM_ (average fraction of open PSII RCs) and R_J_ was observed in the presence of GT (**Figure 7F**). This suggests that GT-caused severe closure of PSII RCs is because of an enhancement of the number of PSII RCs with the Q_B_-site filled with GT.

Obviously, one of the most important primary action sites of GT is perhaps the Q_B_-site of PSII RCs. GT decreases the photosynthetic activity by inhibiting electron flow beyond Q_A_ at the acceptor side of PSII for its binding to the PSII RCs with Q_B_-site.

### GT Binding Niche At the Q_B_-Site of the D1 Protein

Generally, herbicides that target PSII interrupt linear electron transport at the acceptor side of PSII by replacing with native PQ for the Q_B_-site of D1 protein (Oettmeier, 1999). The D1 protein in higher plant is called L-subunit protein in the photosynthetic bacteria. It contains five trans-membrane α-helices and several short nonmembrane helices between the transmembrane helices (Xiong et al., 1996; Kamiya and Shen, 2003). The Q_B_-site just falls between the helices IV and V of the D1 protein from phenylalanine (Phe211) to leucine (Leu275), which is also called the site of PSII herbicide binding (Xiong et al., 1996; Ke, 2001; Trebst, 2008).

**Figure 8:**
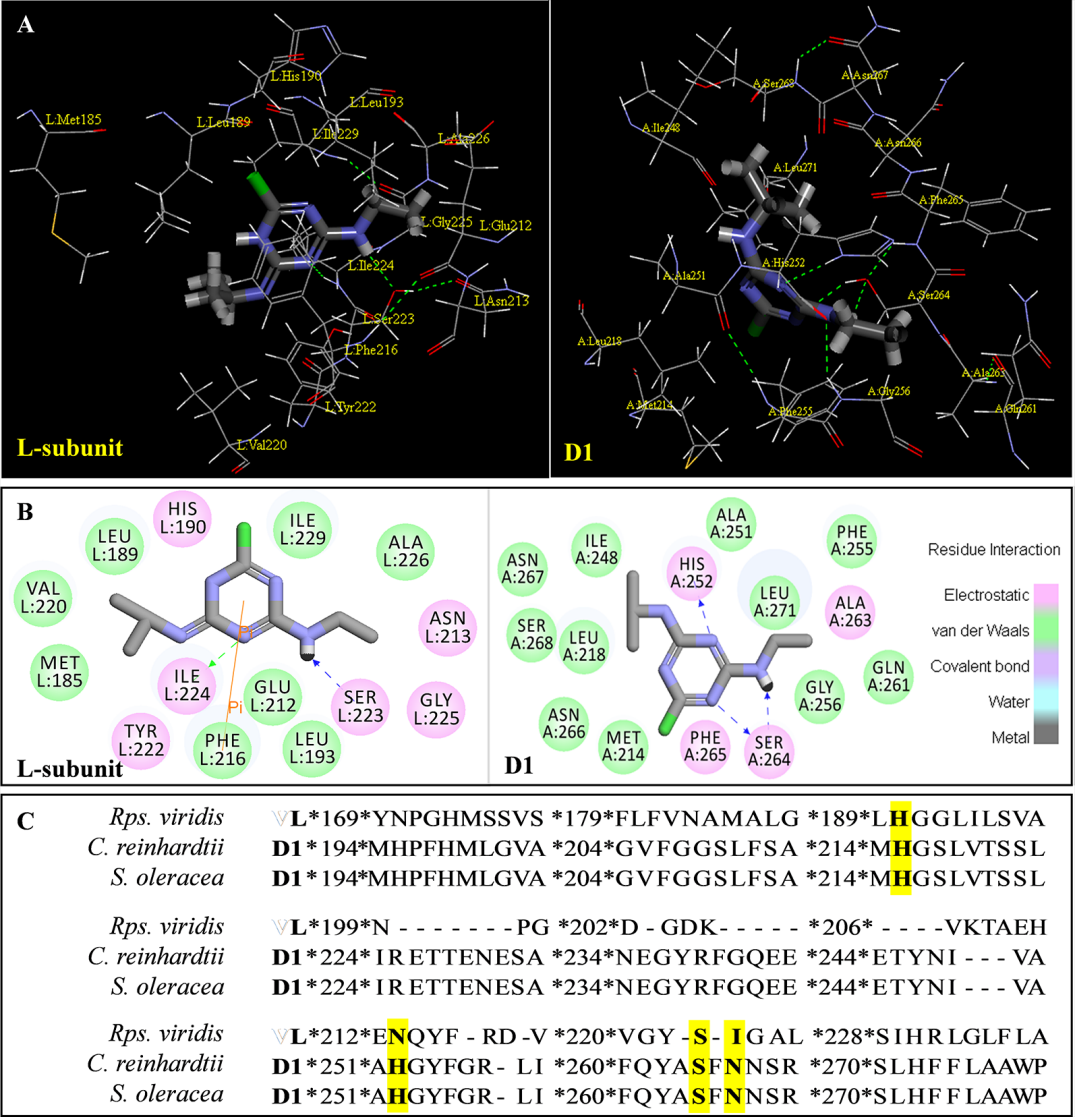
The simulated modeling of atrazine binding to the Q_B_-site. **(A)** Stereo view of atrazine binding environment of the L-subunit of *Rps. viridis* (left) and the D1 protein of *S. oleracea* (right). **(B)** Hydrogen bonding interactions for atrazine binding to the Q_B_ niche. Here, carbon atoms are shown in grey, nitrogen atoms in blue, oxygen in red, chlorine in green, and hydrogen atoms in white. The possible hydrogen bonds are indicated by dashed lines. **(C)** Sequence alignment of the Dl protein of *C. reinhardtii* and *S. oleracea* with the L-subunit of the *Rps. viridis* RC. The bacterial L-subunit sequences are in upper case and the Dl sequence is in lower case.

Above evidence from Chl fluorescence rise kinetics shows that GT may be like DCMU to inhibit PSII electron transfer beyond Q_A_ by occupying the Q_B_-site. In order to get further proof to support this hypothesis, GT was modeled its position in the Q_B_-site at spinach D1 protein using Discovery Studio version 3.5. Meanwhile, to ensure reliability of this method, the simulated modeling of classical herbicide atrazine and DCMU binding to the Q_B_-site was also established based on the available experimental and theoretical data. First, the standard modeling of atrazine to the Q_B_-site was simulated according to the crystal structure information of complexes of the bacterial *Rps. viridis* RC with atrazine (5PRC) and spinach D1 protein (3JCU). It is found that a hydrogen bond can be formed between N-3 of the atrazine ring system and Ile224 of the L-subunit. A second hydrogen bridge between the ethylamino hydrogen (NH) of atrazine and Ser223 of the L-subunit is observed (**Figures 8A, B**, **Table 3**). Furthermore, Phe216 of the L-subunit is involved in atrazine binding by both π-electron systems (**Figures 8A, B**). In addition, four residues, L-His190, L-Asn213, L-Tyr222, and L-Gly225 of the ligand with atrazine are also identified. The results are quite matched with previous studies from X-ray crystal and resistance mutant reports (Oettmeier, 1999; Lancaster and Michel, 1999). **Figure 8C** depicts a homology sequence comparison between the L-subunit from *Rps. viridis* and the D1 protein from *C. reinhardtii* and spinach. The counterparts of L-Phe216, L-Ser223, and L-Ile224 in the L-subunit, as atrazine binding residues, are just D1-Phe255, D1-Ser264, and D1-Asn266 in the D1 protein. Thereinto, D1-Ser264 is the most important for atrazine resistance (Oettmeier, 1999). In modeling of atrazine docking to the D1 protein of spinach (3JCU), D1-Ser264 and D1-His252 are defined as the active sites (**Figures 8A, B**). D1-Ser264 interacting with atrazine is the same as L-Ser223 in the L-subunit. Interestingly, N-1 of the atrazine ring system also provides a weak hydrogen bond to D1-Ser264. Another hydrogen bond is formed between N-3 of the atrazine ring system and D1-His252 not D1-Asn266 corresponding L-Ile224 in the L-subunit.

**Table 3:** Possible hydrogen bonding interactions for atrazine, DCMU and Gliotoxin (GT) binding to the L-subunit of *Rh. viridis* or the D1 protein of *S. oleracea*. The circle refers to the predicted atom position providing hydrogen bond with the indicated amino acid residue.

Com.	Mol. Formula	Chemical Structure	Binding target	Hydrogen Bound Position
atrazine	C_8_H_14_ClN_5_	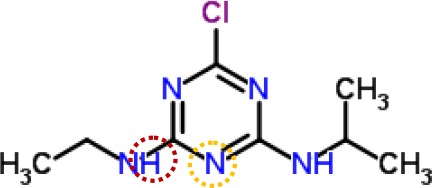	L-subunit	$\begin{array}{c} \text{L}-\text{Ser}223\\ ↓\text{ NH}\\ \text{D}1-\text{Ser}264 \end{array}$	$\begin{array}{c} \text{L}-\text{Ile}224\\ ↓\text{ N}-\\ \text{D}1-\text{Asn}266 \end{array}$
		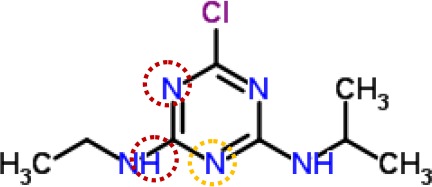	D1	$\begin{array}{c} \text{D}1-\text{His}252\\ \text{N}- \end{array}$	$\begin{array}{r} \text{D}1-\text{Ser}264\\ \text{NH}-,\text{ N}- \end{array}$
DCMU	C_9_H_10_Cl_2_N_2_O	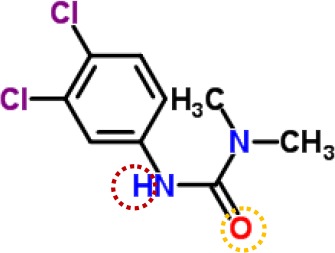	D1	$\begin{array}{c} \text{D}1-\text{His}252\\ =\text{O}- \end{array}$	$\begin{array}{c} \text{D}1-\text{Ser}264\\ \text{NH}- \end{array}$
gliotoxin	C_13_H_14_N_2_O_4_S_2_	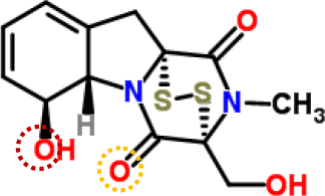	D1	$\begin{array}{c} \text{D}1-\text{His}252\\ =\text{O}- \end{array}$	$\begin{array}{c} \text{D}1-\text{Ser}264\\ \text{HO}- \end{array}$

**Figure 9:**
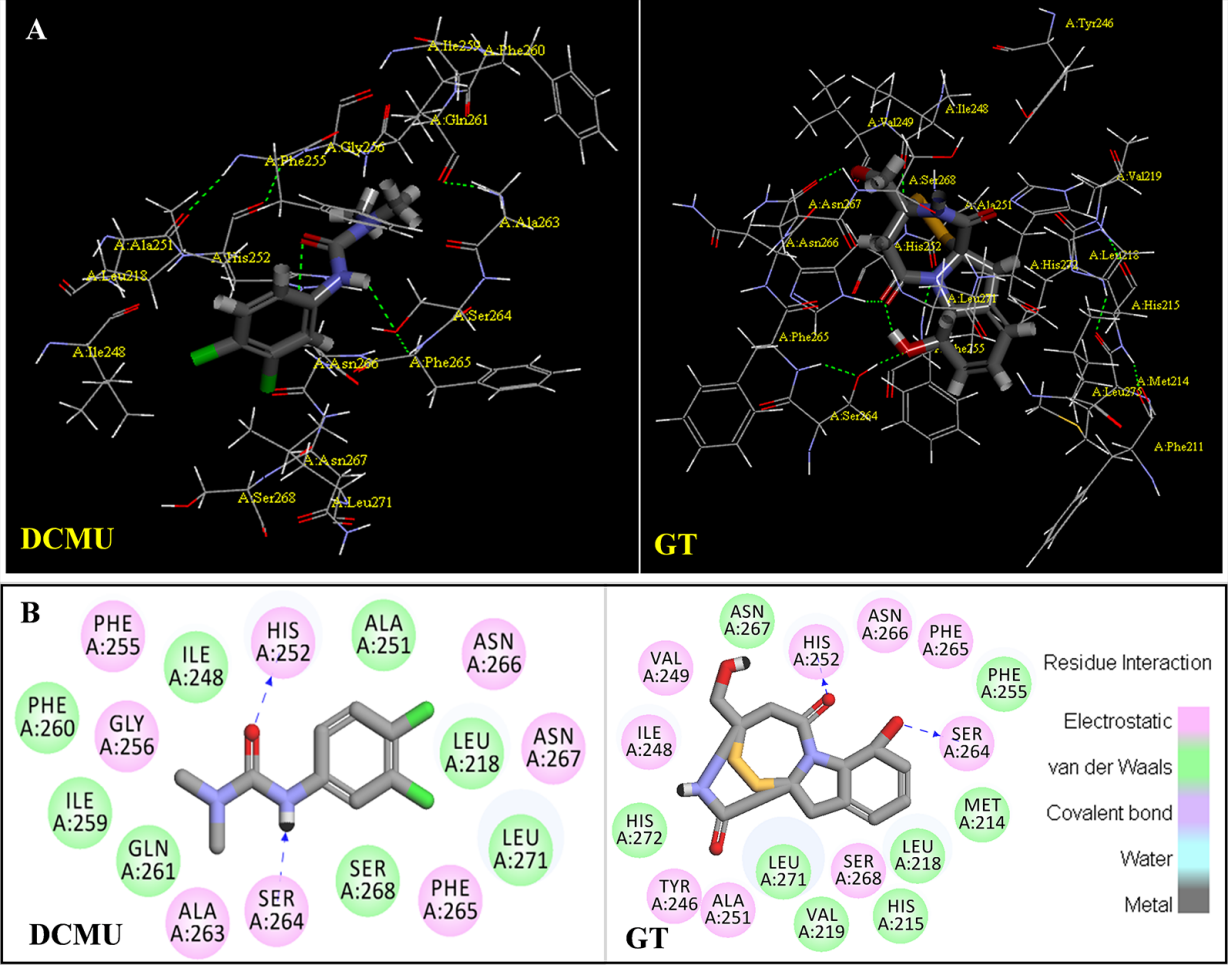
The simulated modeling of DCMU and Gliotoxin (GT) binding to the D1 protein of *S. oleracea*. **(A)** Stereo view of DCMU (left) and GT (right) binding environment of *S. oleracea* D1 protein. **(B)** Hydrogen bonding interactions for DCMU (left) and GT (right) binding to the D1 protein. Here, carbon atoms are shown in grey, nitrogen atoms in blue, oxygen in red, chlorine in green, and hydrogen atoms in white. The possible hydrogen bonds are indicated by dashed lines.

The binding modeling of the D1 protein from spinach (3JCU) docking with DCMU is also presented in **Figure 9**. The D1-Ser264 provides a hydrogen bond to the amide hydrogen of DCMU. Another hydrogen bond is found between the carbonyl group of DCMU and D1-His252 (**Figure 9**, **Table 3**). Previous modeling has shown that the protein binding environment for DCMU is overlapping with that for Q_B_. The residues that appear to coordinate DCMU binding are Dl -Phe211, D1-Met214, D1-His215, D1-Val219, Dl-Phe232, D1-Tyr246, D1-Ala251, D1-His252, D1-Gly256, D1-Ala263, D1-Ser264, D1-Phe265, Dl-Asn266, and D1-Leu271 (Xiong et al., 1996). It is predicted that DCMU orients itself preferentially towards D1-Ser264 by a hydrogen bond (Trebst, 1987; Xiong et al., 1996).

The modeling of GT docking to the Q_B_-site was built on the basis of the available crystal structure alignment of spinach D1 protein (3JCU) through energy minimization and molecular dynamics simulations (**Figure 9**). In the modeling, Dl residues identified to be more likely related to the binding of GT are D1-Tyr246, D1-Ile248, D1-Val249, D1-Ala251, D1-His252, D1-S264, D1-Phe265, Dl-Asn266, and D1-Ser268. Here, it is predicted that hydrogen bonds are formed between D1-Ser264 and the aromatic hydroxyl oxygen of GT, and between D1-His252 and the 4-carbonyl group of GT. This exhibits somewhat different from DCMU. Additionally, the residues, D1-Met214, D1-His215, D1-Leu218, D1Val219, D1-Phe255, D1-Asn267, and D1-His272, are also found within the van der Waals contact sphere with GT. However, to further identify amino acids in the target which participate in GT binding, more experimental data based on the site directed mutant and X-ray structure are needed in the future.

It is well known that all PSII inhibitors share the same binding site on the D1 protein. However, each inhibitor has its characteristic orientation in the D1 protein. PSII inhibitors can be grouped into two families, the classical type with ureas and triazine, and the phenol type with ioxynil and dinoseb (Trebst, 1987). The ureas/triazine family inhibitors have the common structure group N-C = X, where X signifies N or O. They are more closely oriented toward D1-Ser264 in the Q_B_ binding niche. The phenolic inhibitors contain the aromatic hydroxyl group bearing nitro and/or halogen and/or nitrile substituent, binding to the Q_B_-site *via* D1-His215 (Oettmeier et al., 1982; Trebst, 1987). GT belongs to the first family since it possesses the common characteristics group N-C = O like ureas/triazine type PSII inhibitors. The protein-binding environment of GT appears to be consistent with most existing data of the classical PSII inhibitors.

## Conclusions

Above all presentations reveal that GT has excellent herbicidal potentiality attributed to its multiple effects on photosynthetic apparatus. The main action of GT is the arrest of photosynthesis by blocking electron flow beyond Q_A_ at the acceptor side of PSII and then inactivating PSII RCs. The primary direct target of GT is the Q_B_-site of the D1 protein in PSII. Based on the modeling of GT docking to the D1 protein, it is assumed that GT binds to the Q_B_-site by the hydrogen bonds between the aromatic hydroxyl oxygen of GT and the residue D1-Ser264, and between the 4-carbonyl group of GT and the residue D1-His252. It is clear that GT is a novel natural PSII inhibitor with the characteristics group N-C = O. Additionally, GT at high concentration can also inhibit PSI activity by decreasing the reduction of the end acceptors at the PSI acceptor side. So, it is concluded that GT may be an interesting structural framework of a potential photosynthetic inhibitor. However, further studies are needed to clarify and confirm the actual binding site for GT in PSII and PSI.

## Data Availability Statement

All datasets generated for this study are included in the article/**Supplementary Material**.

## Author Contributions

SC and SQ designed research. TGu, JC, YL, HW, YGa, JS, CY, and XW performed experiments. SC, YGu, JC, and RS analyzed data and wrote the paper.

## Conflict of Interest

The authors declare that the research was conducted in the absence of any commercial or financial relationships that could be construed as a potential conflict of interest.

## Supplementary Material

Figure S1 SDS-PAGE analysis of spinach thylakoid polypeptides after GT treatment. Thylakoids with 100 μg Chl ml^−1^ were treated with 1% DMSO, 1 μM DCMU and different concentrations of GT for 0.5 h in the dark at 25 °C. Thylakoid membrane proteins were separated by gel electrophoresis. A total protein containing 15 μg chlorophylls was loaded onto the gel for each sample. SDS-PAGE containing 6 M urea was used with a slab gel containing 4% (stacking) and 12% (resolving) acrylamide. From left to right: maker (Thermo Scientific PageRuler Prestained Protein Ladder, No. 26616) (1), 1% DMSO (mock, 2), 50 μM GT (3), 100 μM GT (4), 200 μM GT (5), 400 μM GT (6), 1 μM DCMU (7) and water (8). CP and OEC indicate Chl a/b binding protein and oxygen evolving complex 33 kD, respectively. The similar result was repeated at least 3 times.
