# Supplementary figures and images for: Novel Action Targets of Natural Product Gliotoxin in Photosynthetic Apparatus
Source: Front Plant Sci. 2020 Jan 17;10:1688. doi: 10.3389/fpls.2019.01688 (PMC6999049; doi:10.3389/fpls.2019.01688)

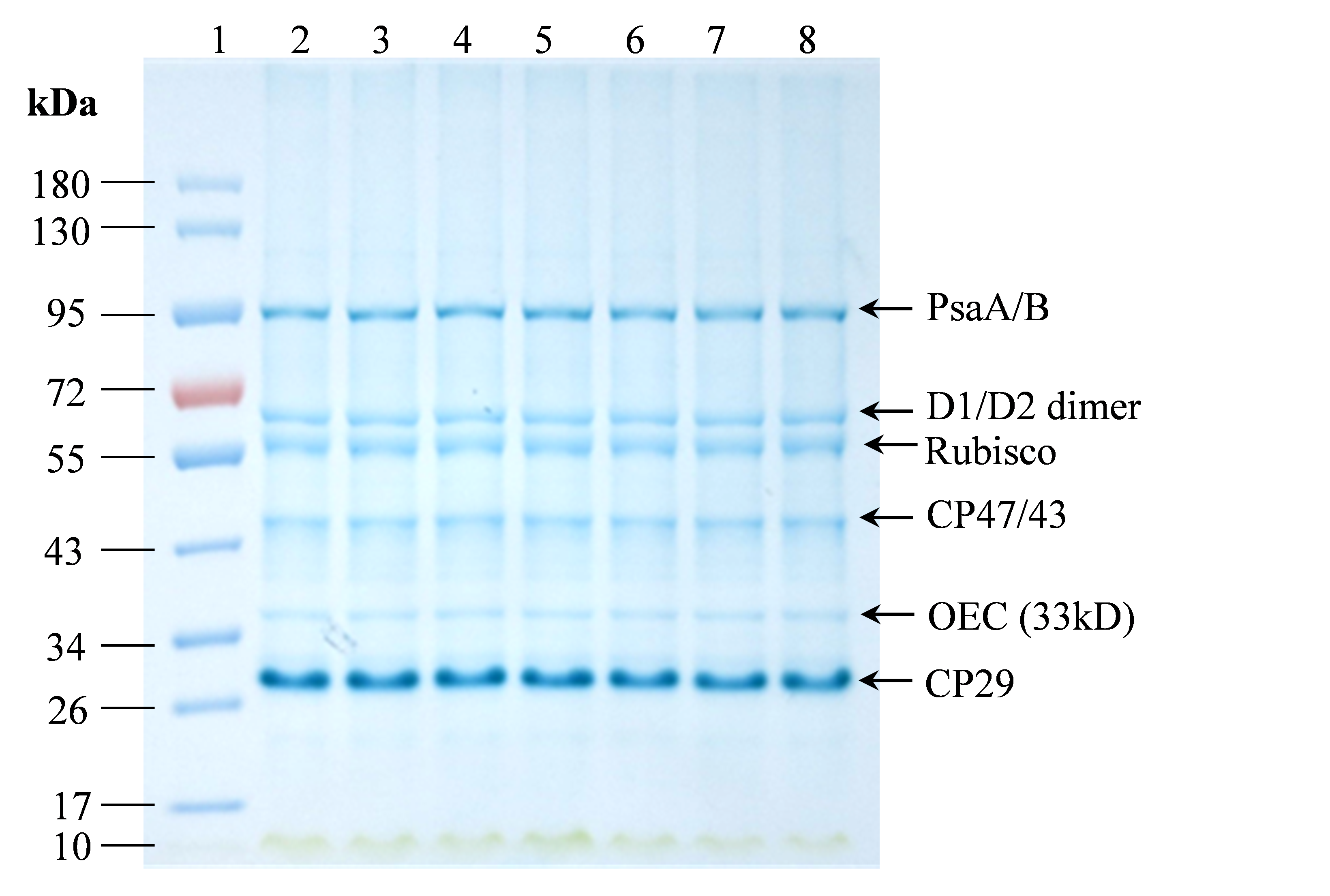

Supplement: Figure S1 — SDS-PAGE analysis of spinach thylakoid polypeptides after GT treatment. Thylakoids with 100 μg Chl ml−1 were treated with 1% DMSO, 1 μM DCMU and different concentrations of GT for 0.5 h in the dark at 25 °C. Thylakoid membrane proteins were separated by gel electrophoresis. A total protein containing 15 μg chlorophylls was loaded onto the gel for each sample. SDS-PAGE containing 6 M urea was used with a slab gel containing 4% (stacking) and 12% (resolving) acrylamide. From left to right: maker (Thermo Scientific PageRuler Prestained Protein Ladder, No. 26616) (1), 1% DMSO (mock, 2), 50 μM GT (3), 100 μM GT (4), 200 μM GT (5), 400 μM GT (6), 1 μM DCMU (7) and water (8). CP and OEC indicate Chl a/b binding protein and oxygen evolving complex 33 kD, respectively. The similar result was repeated at least 3 times. [file Image_1.tif]
